# Socially induced motivation in learning: coping with digital interaction in higher education under the pandemic

**DOI:** 10.1007/s12144-022-03407-1

**Published:** 2022-07-15

**Authors:** Ida Poppe, Lars Erik Kjekshus

**Affiliations:** Department of Sociology and Human Geography, Faculty of Social Sciences, University of Oslo, Moltke Moesvei 31, P.O. Box 1096, 0317 Blindern, Oslo, Norway

**Keywords:** Higher education, Sensemaking, Digital, Motivation, Pandemic

## Abstract

The COVID-19 pandemic resulted in a total digital disruption of all activities at universities. New digital tools and arenas replaced the daily physical interactions between students and professors. How did this affect motivation and learning outcomes? This article uses the pandemic as a prism to understand how and why social relations and interaction are important in the educational system. Data were obtained from 26 informants in two case studies (study programs). A total of 12 in-depth interviews with employees and 4 group interviews with 14 students were performed at Oslo University during the pandemic (2020–2021). We explore an alternative understanding of social ties in relation to the educational process and the importance of social interaction in sensemaking and self-determination theory concepts. As digital disruption creates a social disconnect for most actors, it becomes prevalent that social activity, both formal and informal, seems to be an important source of motivation for both students and faculty members at the university. We introduce the concept of socially induced motivation as an important aspect of learning. The tendencies in the informants’ accounts of the social interaction are perceived in this context as sensemaking the university as an organization and how it solves its missions and assignments. Socially induced motivation is an important concept, both in relation to *work* in general and specifically to work in higher education. Our study shows why universities should strive to facilitate socially induced motivation in the future.

## Introduction

Overnight, on the 12th of March 2020, all communications and teaching at the University of Oslo (UiO) were transformed into digital solutions. “Zoom” in the living room became the new auditorium, and everyone did the best they could to get things going. In retrospect, the process has been said to have exceeded all expectations when extreme reorganization occurred because of the coronavirus pandemic. From the 12th of March 2020 to the 18th of May 2020, all the university’s premises were closed, and much of the teaching took place on digital surfaces with digital home exams. As a result of these changes, several technical functions had to be developed and adapted for full functionality (UiO 2021a). Enormous resources have been mobilized to put in place both the technical tools and the expertise that must come with them. The development of the period is thought to have a significant effect on the digital build-up of both material and social resources in the organization.

In this article, we discuss how digitalization has affected motivation and possible learning outcomes. The coronavirus pandemic represents an unusual situation and a kind of natural experiment in which socialization is excluded as a factor in learning. This enables us to study the importance of socialization in learning, as we can observe how learning and teaching are unfolding under the pandemic. Our hypothesis is that when the social field and its functions was severely limited during the pandemic, it also has consequences for the effectiveness of the functions of formal practice.

Since the pandemic, both the university, academy and the media have devoted much attention to assessing how the changes affect students. Key topics in this period have been discussions about mental health, financial challenges, and positive and negative aspects associated with digital teaching (Aucejo et al., 2020; Bao, 2020). A literature review in Current Psychology conclude that *“higher education institutions and governments should take action to ensure the safety and the physical, social, and mental wellbeing of the students”* (Jehi et al., 2022). Persons at risk for suicide usually approached suicide through searching information and news regarding self-harm and suicidal behaviors on Internet (Solano et al., 2016) and measures of affective temperament-types were independently and more strongly associated with lifetime suicide attempts than was diagnosis of a major affective disorders or other variables (Baldessarini et al., 2017). Studies also showing the risk of burnout and fatigue for student during the Covid-19 pandemic (Moroń et al. 2021; Hassan et al., 2022). Whereas some would say that physical education is extremely important for learning outcomes and well-being, others point out that increased digitalization is important for education’s accessibility for all (Strand, 2021; Torgersen, 2021) and the need to better understand online teaching and learning during the COVID-19 pandemic (Kumar et al. 2022; Chan et al. 2021; Pandian et al. 2021). At the same time, studies from England show that digitalization triggered by the period has a negative effect on university employees’ pedagogical roles and personal lives (Watermeyer et al., 2021) as in the US (Pressley & Ha, 2021). Studies of students experience of online teaching and learning has shown a complex set of factors of institutional and pedagogical responses as well as individual factors (Bisht et al., 2022; Flores et al., 2021; Osman, 2020).

This article provides insight into how students and staff at a university has handled digital interaction during the pandemic and an analysis of the impact of de-socialization during the period. First, we provide a description of our theoretical perspectives, with a special emphasis on sensemaking, social interaction and motivations, and their possible importance for learning and teaching. We present the most important findings in the analysis section. These findings are further discussed in the last part of the article, with suggestions for further steps for the university to encounter these issues in future learning and teaching.

## Theory

Our main argument is to enhance the importance of socialization in teaching to not only view socialization as a side activity happening in students’ spare time but also acknowledge socialization as an important variable in teaching and in the transformation process from teaching to learning. The lack of social interaction in teaching affects motivation and, eventually, the outcome of learning.

To understand these processes, we built our theoretical approach on the concept of sensemaking and the work of Karl Weick. The concept of sensemaking was first introduced in the understanding of crisis handling (Weick, 1990). The concept of sensemaking is fundamentally social constructivist. How we give actions meaning is essential for understanding organizational outcomes, processes, interpretations, and change (Leonardi & Barley, 2010; Thornton et al., 2012; Weick et al., 2005). Associated with role understanding, mental forms, and established problem solving, the individuals in the field act efficiently and purposefully with the help of sensemaking. In the face of deviating situations or problems, one tries to solve them by applying existing knowledge and experiences. In the first instance, one tries to solve the task as similarly as one would otherwise do by drawing on existing knowledge; however, in the face of major problems, one is potentially forced to improvise. Sensemaking is a retrospective mental and social process that rationalizes actions in organizations. In many ways, the process deals with how one “speaks an event to life” to ascribe meaning and “make sense” of what has happened (Thornton et al., 2012, p. 96; Weick et al., 2005, p. 409). Change in organizations can thus be both a result of and a trigger for sensemaking processes. In this way, the sensemaking process deals with the interplay between action, interpretation, negotiation, and, to some extent, learning (Weick et al., 2005, p. 409). In the context of this study, the problem-oriented sensemaking that arises because of the coronavirus pandemic is particularly relevant and will provide a deeper understanding of the role of social interaction in teaching.

**Fig. 1:**
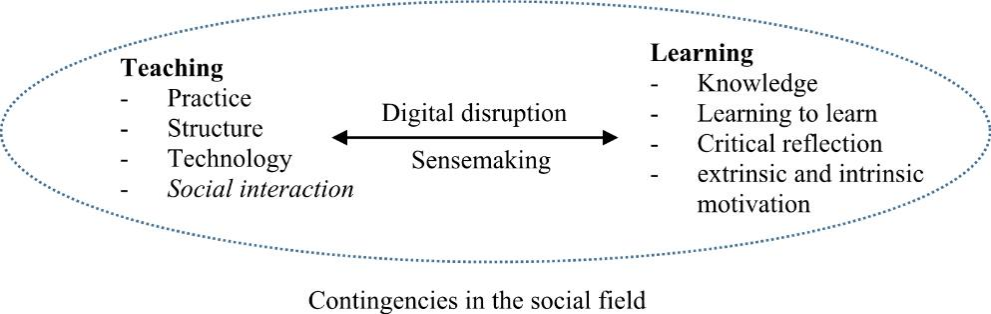
A conceptual model of sensemaking and the importance of social interaction in teaching for the outcome of learning

The main goals of the university are teaching, research, and dissemination. The outcome of teaching is students’ learning seen as knowledge, the ability to learn, critical reflection, and academic motivation. The process of learning is supported by the structure, technology, and practice of teaching, all happening in a more or less conscious social field. The content in the social field is directly dependent on an organization’s formal and material existence and is largely based on social interaction. Figure 1 summarizes our main arguments.

Social interaction can be defined as a communicative interaction between two or more parties. This can consist of verbal communication and nonverbal communication (Alcock & Sadava, 2014). In connection with social fields, social actors are thought to acquire knowledge and the ability to participate in interactions that may be specific to the field in question (Thornton et al., 2012). For example, in the open discussion, the seminar, discussion, and interaction are conducted as a basis for knowledge development (Bennich-Bjorkman, 2016; Tjora, 2019). The social field influences the interaction at the university and produces a culture that the students acquire as part of the socialization process in education.

Motivation is the explanandum variable in this study (Ames, 1992), and self-determination theory is an interesting perspective in this context. This is a social psychological perspective that is used to understand how motivation works when it is associated with work and how one can facilitate the strengthening of motivation in organizations (Gagné & Deci, 2005; Ryan & Deci, 2000). Self-determination theory distinguishes between intrinsic and extrinsic motivation and emphasizes three *needs* that must be met if one wants to foster intrinsic motivation in the workplace and facilitate the internalization process (Brahm et al., 2017; Kickert et al., 2021). Here, they present social communities in a workplace as a basic psychological need that is particularly crucial for the internalization of extrinsically motivated practices. The other two needs mainly have a positive effect on intrinsic motivation and are based on the fact that actors want to feel *competent and autonomous* in the work they do (Gagné & Deci, 2005). Environments that facilitate these three needs thus facilitate actors with strong intrinsic motivation and full internalization of extrinsically rooted motivation. The social context is therefore presented as fundamentally valuable and crucial for the socialization process by new actors in the field (Gagné & Deci, 2005).

Bogler & Somech (2002) explicitly discussed the connection between students’ motivation and how they relate to socialization in the field. They emphasized the distinction between extrinsic motivators for graduating and the status that accompanies this, and the intrinsic motivation for acquiring knowledge and expanding one’s own horizons. Students’ socialization tactics are decisive for whether they thrive and how well they perform at university (Bogler & Somech, 2002). In other words, regardless of the motivation for participating in the field, socialization processes and the acquisition of local culture and knowledge are presented as central to success.

The pandemic and digitalization of teaching enable us to study these aspects of socialization further, as the lack of social interaction is evident. By studying sensemaking processes in the context of the pandemic, we observe the importance of social interaction for learning and motivation.

## Method

The empirical data were derived from studying two bachelor programs at UiO. Most of the teaching is organized in lectures and seminars. UiO is a broad university covering a plethora of research fields and study programs, and the organization is complex and decentralized. UiO consists of almost 27,000 students, almost 7,000 employees (UiO, 2021b).

The two bachelor programs were chosen, aiming to explore the university as such, but through the lens of two fairly different programs. One from the Faculty of Social Sciences (SV) and the other from the Faculty of Mathematics and Natural Sciences (MN). We assumed that different academic disciplines would have different needs in relation to work and study environment, mostly materially but also socially. The idea was that the similarities shown between the two cases would provide a more comprehensive understanding of the effects of the material and social disconnection that characterizes the university during the pandemic lockdown period.

Data were obtained from 26 informants at the UiO, all with an affiliation to the two chosen study programs. The study’s informants were both academic staff, including course coordinators; administrative staff at the study program, department, and faculty levels; and a group of students from the selected studies. The study was conducted via semi-structured, in-depth interviews with 12 employees. The interviews with 14 students were conducted as group interviews, all with three to four participants of different study levels who were affiliated with the same bachelor program.

Many of the employees who participated in the interviews were recruited directly via email addresses, which were available on the university’s homepages. In advance, we went through the schedules and their role descriptions to check that they were relevant informants. This process was partially successful and ensured the conduct of about half of the employee interviews. The rest of the employees were referred via contact persons in the administration. Contact with the students was established using Facebook groups, joint emails from the administration, and a variation of the snowball method (Tjora, 2021), where we got help from both course leaders and individual students.

The coding of the data was thematic, combining both inductive and deductive reasoning, starting inductively with the empirical data (Tjora, 2021, p. 175). A starting point for the coding and analysis was based on fully reading all the interviews, aimed at obtaining the study’s empirical tendencies and thematic content. Later, the “first impression” of the empirical content made way for the first round of sorting-based coding for further systematization of the data. The work was performed manually using color coding and comments in the documents. The theoretical framework was chosen after the actual data collection. From this, among other things, the interest in motivation in connection with the study’s topics grew out of the empirical data and not of a preexisting theoretical interest. With the alternation between the empiric-oriented approach and theoretical connections along the way, motivation presented itself as a central aspect in the understanding of the university as a social field and value-producing organization.

## Analysis

In our analysis, we aimed to obtain insight into how students and staff were coping with digital transformation and how it affected learning and teaching. Three central topics were determined to be especially relevant. First is the experience of how the boundaries of the public versus the private space were disturbed and became blurred. Second is experiencing Zoom fatigue and quality loss when the interaction is digital. Third is the lack of informal arenas and the important functions of these arenas.

### Blurring of public and private spaces

As a result of the coronavirus pandemic and the introduction of comprehensive infection control, the practice from the physically established campus has moved into digital solutions and the private space. Material boundaries ceased. The lecture hall, meeting room, and seminar room were replaced by the digital meeting tool Zoom. In the pandemic, the only channel for contact with the university and the networks was digital. When things were torn away from their original form, the informants perceived work and study as more fluid and indistinct. An example is the understanding of normal working hours.

#### Staff

Borders and barriers ceased, students called on a Sunday, but there was something about the whole situation. […] as it became very difficult to distinguish between work and family life, because you had to do things when you had time.

Similar quotes also come from the students who talk about the difficulty of staying focused on studies when they are in the “wrong place”. Some of the students expressed that they would previously have thought that more flexible teaching would have been a positive option, but no longer when the freedom of choice was removed. Several also expressed that dishes, cooking, or Netflix in a new way compete with teaching time. When the practice that previously existed on the university’s premises was moved into the home that was generally associated with other practices, staying focused became more demanding.

#### Student

I think it’s something when you’re not in the physical space. When you sit in a lecture hall, you are there to listen to the lecture, but when you sit in the kitchen, the surroundings in a way indicate that you should actually do other things. And how long should you do it before your brain gets used to “here it is studied”.

The difference between the two spheres is sensitized and redefined. Students are becoming aware of a separate problem in the teaching practice entering the *private spheres*. Many students choose not to have their cameras on in class unless they must. Some factors that are mentioned in this context are “the dishes in the background,” “sitting unkempt in bed,” “roommates in the same room,” and the like. In addition to these situational elements, feeling observed by other participants also seems to be a source of general discomfort. This is referred to as a sense of loss of control.

The academic staff problematized the transition to the digital, particularly with a lack of interaction in teaching. At the same time, professionals emphasized that the establishment of better digital communication and video tools may make it easier with international cooperation. Some of the students also associated digital arenas with positive opportunities, especially if they were offered hybrid tuition. Aspects that were emphasized include saving time when traveling for those who live far away from campus. Students pointed out that it provides a completely different opportunity to balance everyday life better in terms of sleep, food, work, and leisure. The fact that lectures are made available as digital recordings also increases the flexibility of where, when, and how many times one can use the lectures.

### Zoom fatigue: Interaction in the digital

The interaction is affected by moving to digital surfaces, a change that comes in addition to the practical consequences of the home office. Digital interaction is demanding. Clumsy communication, lack of norms, and a sense of distance are mentioned as the results of the tool’s limitations. A dialogue at Zoom involves technical challenges, such as delay in sound or unstable internet, and social challenges, such as lack of nonverbal communication. At the extreme, this means that good interaction does not fit into digital teaching.

The limitations that the study’s informants expressed around the digital forms reflect familiar issues from the digital interview process itself. The interaction lacks flow and staying actively involved in the interaction is more difficult. The tool is presented as “a patch on the wound” by several, a formulation that emphasizes these technical and social limitations.

#### Staff

You can say a lot of positive things about Zoom lectures, that people from all over the world can follow it, you can almost sit in Bali to follow a lecture if you want, but it is not an optimal lecture or group teaching. So, it was a patch… The advantage is that you do not have to show up [physically], but you do not get the sense of community in it.

In digital environments, formal activity can be maintained, but it is more demanding when it comes to establishing social relations and the experience of a community. The students emphasized the problems, especially in connection with seminars and how much more difficult it was to have a good and active discussion. The prerequisites for a good interaction seem to be that one has a pre-digital relationship, that the group is not too large, and that everyone commits to being actively involved in the conversation. These perceptions are confirmed by employees. They pointed out that collaboration in small groups and with well-known colleagues can still work well. Digital interaction *can* thus work well when it is arranged for it, but the practice is still perceived as more demanding than physical meetings. A recurring theme from the interviews is that one simply becomes more tired of participating and carrying out activities, such as meetings and teaching, on digital surfaces, a form of “Zoom fatigue”.

#### Staff

(…) I talked to people about it already in April-May, Zoom-fatigue, that people are simply completely exhausted by it, to meet digitally. Because you do not get the reaction patterns of people, you do not get any feedback, and I notice it myself, you are completely boiled in the head by having home office and Zoom meetings.

Interactions that previously could have been effortless become far more demanding in digital because you do not have the opportunity to “read” those you interact with. The fact that interaction and practice become extra demanding can be linked to the challenge of actively maintaining focus, which was mentioned in the previous section. In addition, the challenges are thought to be a direct result of technical limitations in the digital and the lack of the non-verbal interaction.

#### Student 1

It becomes difficult when we are asked questions, because when it was physical you could look at each other and see who is going to talk. For there is much more gaze and body language and so on, but when it is not there, it is very difficult.

#### Student 2

Yes, it’s also so easy to talk in each other’s mouths because there’s a delay. Two people can start talking at the same time, but you do not hear it because it causes [delays], then it becomes like that . yes, no, eh, uhm, eh . no one can start talking, and it happens often.

These quotes reflect how the digital format can limit interaction, both verbally and nonverbally. Several informants also pointed out that digital teaching also affects motivation. In a “normal” interactive situation at the university, one can use one’s own communication, hearing, and vision to handle the situation. The interaction has a flow because everyone involved is able to read the room and therefore also participate in accordance with unspoken social norms for the conversation. When the interaction is transferred to the digital, one first loses the opportunity to *see* everyone involved. In addition, one loses the opportunity to *hear* when the conversation has an opening because of technical elements, such as delay and chopping in sound.

Furthermore, in larger gatherings, such as lectures, the interaction is strongly influenced by the limitations of the digital format. One aspect that becomes very prominent in this context is that lectures, as a form of teaching, which are otherwise often criticized for the students’ passive listening to the lecturer’s monologue, also have basic elements of interaction. Staff refer to giving lectures as a social situation that can give them a bit of a rush, and that it is in this context that they get the opportunity to interact with the students. Several of the academics expressed that although interaction in a lecture does not necessarily consist of discussion and conversation, it is perceived as an interactive situation in the interaction between one’s own presentation and the students’ nonverbal feedback. The lack of interaction in the digital format therefore entails difficulties in maintaining a degree of enthusiasm and commitment that they are used to being able to maintain in a physical lecture. Traditional lectures are explicitly compared to a feeling of standing on a stage, not necessarily to get applause when one is finished but to experience the presence and reactions of an audience.

#### Staff

In a regular lecture, you follow how people have their eyes and facial expressions and all that, we capture much more of it than the students are aware of. It’s almost as with actors who say they need the audience; we need to get some response!

The students also seemed to miss part of the interaction that occurred in a physical lecture. When one is no longer present in the same physical space, it is to “raise one’s hand” to ask a question something completely different, whether there is an opportunity for it at all. Staff have received far more questions in chat functions or other messaging services during this period, which one of them associated with “that the teaching has moved into the students’ digital home field”. The combination of a continuous digital question thread and difficulties in seeing and interpreting the audience results in a particularly demanding situation for those who teach. The students thought that the opportunity to ask questions in writing lowered the threshold for asking questions in the digital form, but the result is not always desirable.

#### Student

Yes, there is a lower threshold for asking questions in the chat, but that entails one of two things. Either the question disappears in the masses, or it can with some lecturers mean that they completely derail and do not return to what they were supposed to go through.

The students pointed out that asking questions in writing is far less “accurate” and that the answers the academic staff give in writing are often misunderstood. This tendency can be linked to the fact that the flow one usually finds in good interaction is partly dependent on the immediate interaction and that one can express a reaction or question to what is being said when it is being said. When questions are presented independent of the original reasoning, the question loses part of the context and thus also part of the content. The interaction and questions in the teaching thus lose momentum in the digital, so that much of the information in the interaction is lost. Taken together, these factors seem to mean that engagement and questions in teaching are limited in digital format.

### Lack of informal arenas and spontaneous interactions

A threshold that is also changing in the digital world is general attendance and active participation in teaching. This topic is highlighted by both employees and students. Some students pointed out that when a digital lecture or seminar is perceived as unproductive, the threshold for pressing *leave meeting* is much lower than it is to get up and leave a lecture hall. At the same time, the detachment from the physical space and the total and immediate end of the interaction are pointed out to be unsatisfactory. This can be interpreted as a social vacuum when the Zoom session ends. This is reflected in the following quote: “Even when you have finished a lecture or seminar, it’s just like that, *leave meeting*, then you sit there all alone”.

As indicated in the theoretical framework, the university is assumed to be both a formalized organization and a social field. The university is a meeting place where informal activity is highly valued and more functions are assigned. By informal activity, we mean all activities that are not administered by the university. This includes meeting colleagues and students in the hallways and having the opportunity for spontaneous dialogue and information sharing. Studying or working at the university during the coronavirus pandemic is perceived as less satisfying.

The academic staff pointed out that the perceived increase in written questions to the lecturer may be a result of the students not meeting at the university and talking to each other. This assumption was confirmed by the students’ descriptions, and this tendency is reflected in the following quote.

#### Student

Yes, the good old discussion, it does not exist, and I have noticed it used to help me, when you get to talk about things. That you can do it during a break or whatever, but you do not get it now, and it is such a great way to learn.

“The good old discussion” refers to something that one would otherwise get in informal interactions that occur when people are physically gathered at the university. Spontaneous social interactions seem to trigger knowledge development and information sharing. The fact that the students do not want to ask oral questions in the digital context, that the answers to the written questions are often imprecise, and that the students do not meet at the university either, results in some of the support the students have traditionally had access to being limited in several ways.

For example, the mobilization of colloquium groups as separate “learning organizations” may be considered crucial for some students, as they receive few follow-ups and are dependent on continuously building new relationships for this purpose. Some courses do not even have seminars, so the colloquium and the interaction with fellow students become particularly central. A smaller group, either in the form of colloquium groups or close friendships, is a place where many students find opportunities for support, information sharing, and joint knowledge development. Variations occur in how stable these relationships are, but the common denominator is that in this type of relationship, one can ask questions, discuss, and gain a better understanding without reservations. Some of the students who did not already have established relationships of this type expressed difficulties in establishing them during this period of digital teaching.

#### Student 1

It’s even harder to get a colloquium group, because the people you had a nice conversation within class just disappear [when the class ends].

#### Student 2

Yes, you may walk past them in the hallway and suddenly dare not say hello, because they are strangers. If the first conversation had been physical, this would not have happened.

The quotes illustrate how important physical presence at the university is to build the kinds of relationships and functions that students seem to find in smaller groups. One of the students who discussed these issues in the interview said that she had tried to ask fellow students if they wanted to form a colloquium group with her.

#### Student

I tried to do something nice and ask in a lecture with 300 students if anyone was interested in being in a colloquium group with me, I got over 250 answers. In the end, someone pointed out that I could set up a group on Facebook where everyone could be added… And it just turned out to be way too much work.

The response to her inquiry was an astounding number of messages from her co-students, a tendency that may very well be linked to the loss of informal arenas that would naturally facilitate this function. A prominent tendency exists in several of the informants’ statements: Digital teaching does not facilitate the type of interaction that usually characterizes the university. Then, it is both a question of the basis for stable and secure relationships and the spontaneous interaction and discussion that usually occurs when one has access to campus as a meeting place. The students’ time at the university is referred to as a period for joint exploration, where meeting and spending time with other students is crucial in the meeting with the university as an institution. Professional, social, cultural, and practical competence must be acquired. The typical example used when the informants talk about these things is the general “to meet and talk to people in the corridors,” as if it is in the corridors where part of the university’s practice is located. The university itself, as an organization both materially and socially, makes it possible, but processes take place in informal interaction, and the smaller social groups are valuable and important.

## Discussion

Although the digital arena enables faster and more frequent meetings, our main finding is how a mental discrepancy emerges between place and activity that makes maintaining concentration through digital teaching at home more demanding. Also, the information exchange happening in the digital arena is experienced as thin and without the information richness the physical encounter enables. Communication is poorer when students and staff are not able to meet physically, and this affects the processes of sensemaking in learning.

In self-determination theory, the individual’s ability to feel competent and autonomous in the work they do is crucial for intrinsic motivation (Gagné & Deci, 2005, p. 337). Considering this understanding, it will have little to say whether one is both inspired and engaged in the field when attempts at engagement and questions encounter problems both in technical limitations and social interaction. At the same time, one does not have the opportunity to seek confirmation or fellowship from other students or from a lecturer when the interaction does not work. Being the one who asks questions that derails the lecturer and at the same time does not have the opportunity to correct misunderstandings may mean that even a normally actively participating student is passivated.

A lack of social interaction can seem to affect the motivation of the students. When one does not spend time physically at the university, social interaction is reduced. Social interaction in which a normal situation seems to be a driver of visiting the university and the idea of being *a student*. The consequence is that one loses informal information sharing, good discussions, and, in this way, part of informal knowledge development. In the same context, the students also expressed their difficulty in building secure relationships with other students, which is perceived as crucial for part of the students’ development. Therefore, not all functions are maintained, even if teaching is carried out and students attend exams. The students’ experience of the transition to digital teaching is that the shift has a negative impact on focus and the opportunity for interaction and motivation—elements that can have an effect on both learning outcomes and general well-being.

What is most clearly reflected in the informants’ reasoning and problematization of the coronavirus pandemic is the focus on the limitations of the social field. Informants from both the student and employee groups discussed a lack of the social field. The digital interaction of the period is expressed as demanding, and all express a concern for the students’ development without access to campus.

The social field may seem to represent a necessity for some students and their roles at the university. Compared with the staff, the students are thought to have a far less formalized role. One does not get much follow-up; hence, the extrinsic incentives for motivation become both fewer and weaker. In subjects with compulsory activities, not attending lectures or actively participating in seminars can have consequences, but in many courses, this is also voluntary. The students receive a grade at the end of the semester, but beyond this, the role of the students is relatively independent in relation to the university as an organization. When the students themselves are responsible for getting through their education and taking their exams, with very little external control and follow-up, the socialization becomes even more important for the students to succeed and be able to achieve a degree.

The tendency to focus on the social field is probably given that it is this part of university practice that is missing in the transition to a digital format. On the one hand, as Gagne and Deci (2005, p. 337) indicated, socialization is a basic psychological need for well-being in the workplace and hence is important for all roles in an organization. On the other hand, there seems to be something about social interaction at the university that is also motivating, especially for the academic staff and students. In other words, it has social functions in the organization that extend beyond just job satisfaction.

Independence and responsibility for one’s own learning are basic expectations set for the students, and the social aspect proves to be crucial in this context in this study’s analysis. Given that during the pandemic, one does not have access to the meeting place the university represents, it clearly goes beyond the possibility of good interaction in teaching, and the spontaneous interaction in the social field disappears. As a result, parts of the students’ socialization process are also disrupted.

How study programs are designed, how much compulsory activity is associated with a course of study, and how stable student groups are, are thought to be relevant variables that affect both the process and the outcome of teaching. A difference between MN and SV can be assumed to be directly related to the fact that SV students belong to larger and more volatile student groups and that some of their courses do not necessarily include seminars or group teaching. In addition to traits in the environment, students’ personality traits and motivation to participate in the field can be decisive factors in what a socialization process looks like. Bogler & Somech (2002) showed in their study of students’ socialization processes that the initial motivation to participate in the field will influence which socialization tactics students use. Not all students use the social field and are active in the student community. At the same time, socialization is thought to be a common denominator for those who do well at university (Bogler & Somech, 2002), not necessarily because one acquires the student culture, but because one acquires a part of the university culture.

Socialization is the basis for developing an academic identity and thus becomes both a basis for belonging and a tool for success in the field. One can therefore argue that the social field is an important supplement to formal teaching practice. The reservation some students express to ask questions in a lecture hall and hence also in the digital can be compensated for in social activity. Where digital and as far as traditional teaching has limitations for interaction, social interaction in a normal situation can compensate for any shortcomings. As the coronavirus pandemic limits both formal education and the social field, a predominant risk may be that more people will receive lower learning outcomes, not only related to the academic but also to the level of competence in a broader sense—the social and cultural competence that is achieved with a comprehensive education.

In social interaction, secure relationships and knowledge are built through joint sensemaking. One “finds out” the system and produces one’s own identity at the university through these interactions in the seminar, the colloquium, parties on the weekends, and the general student life. The lack of access to the social field, which causes limited opportunities to form colloquia, get to know other students, and build secure relationships, is something that is problematized. University students are thought to be given a high degree of autonomy and, hence, little external control and a need for self-regulation. By establishing and interacting in smaller groups or close relationships with individuals, some students also intend to establish their own drivers and motivators for success in the field. Therefore, we will refer to *socially induced motivation* as relevant to the activity in the field.

Socially induced motivation is reflected in two different conditions discussed by the informants in this study. The first source of socially induced motivation is related to formal practice at the university. Being in the same room, getting verbal and nonverbal feedback in the interaction, discussing, and conducting joint knowledge development seem to be motivating factors for both students and staff. Among other things, the academic staff who give lectures emphasize that interaction with students in a lecture hall is often an activity they value highly in their role. References are drawn to be on a stage, and the social interaction is suggested to be a source of enthusiasm, drive, and motivation outside the lecture hall. This first form of socially induced motivation can be linked to the energy one gets from good and effective interaction, where the information exchange is rich and thick (Ogara et al., 2014)—interaction that in a normal physical situation is facilitated in the formal practice at the university.

The second form of socially induced motivation is within the informal social field, the university as a meeting place, and the activity that arises there. It is informal meetings with colleagues, fellow students, and others on the university’s premises that can act as drivers for being active in the field. Whether this interaction consists of short private conversations or professional discussion and information sharing does not seem to be decisive; rather, the possibilities for different conversations are associated with something attractive. This type of social interaction thus represents both a break and work in everyday study and work, as these meetings can consciously or unconsciously affect professional development. As Gagné & Deci (2005) emphasized that social interaction is a basic psychological need, we believe in the context of this study that it also represents something more than that, as social interaction also has central functions that can be linked to the university’s value production.

Considering self-determination theory, social motivation can be thought to be rooted in both extrinsic and intrinsic motivation (Gagné & Deci, 2005). Social activity is not explicitly associated with an exogenous reward or punishment. But some students seem to tactically mobilize social relations to colloquium groups and knowledge development, it can still be considered an activity that is targeted in terms of professional development and, in the long run, also good results. In other words, social activity is an action that can be extrinsically motivated to succeed in the field. At the same time, the social interaction seemed to be regarded as fundamentally motivating by informants, then as intrinsic motivation. Among these, one can imagine that individual differences occur, but the point is that social interaction in the long run is also established as a working method in professional development, an activity that is therefore also internalized and associated with autonomous motivation in the field. This is in addition to the fact that it represents a basic psychological need for well-being and the internalization process itself (Gangé & Deci, 2005).

Those who hold roles as professional employees can be considered already socialized actors in the social field. These individuals are already shaped by the university and can be assumed to be highly competent actors who are no longer as *dependent* on social relations to assert themselves in the culture (Friedland, 2009). One can then imagine that this group is intrinsically motivated by professional interest and extrinsically motivated by salary and recognition, which they are also considered to be. However, when they also emphasize social interaction to the same extent as the students, it tells us something about how fundamental the social field can be in the exercise of academic roles as well. There are functions in the social field that one fails to maintain at the same level during the coronavirus pandemic. The formal organization, practice, and technical structures can be partly translated into the digital, but not the functions that lie in the informal social interaction and hence the information richness as such.

By taking this into account, one can imagine three key motivators for academic staff and students: extrinsic motivators such as gain recognition, good grades, and salary. Intrinsic motivators are genuine professional interest and enjoyment of knowledge development. As determined in this study, socially induced motivation may be rooted in both extrinsic and intrinsic justifications, but seems *autonomous* for several of the actors (Gagné & Deci, 2005). Whether this autonomy is justified by intrinsic anchoring or internalized extrinsic motivation is not considered decisive for the relevance of the concept.

The study’s findings suggest that the university, as a social meeting place, is a fundamental part of the organization, almost as important as a lecture, a seminar, or an exam. When social interaction has such central functions for both professional and individual development and when the socialization process itself is a basic function in the field, the socialization also becomes motivating in itself. Social interaction is internalized as part of the working conditions, and when this part disappears and the interaction becomes of poorer quality, it also seems to affect the motivation of staff and students.

The tendencies in the informants’ accounts of the social interaction are perceived as sensemaking the university as an organization and how it solves its missions and assignments. The social field seems to be crucial for the university’s teaching, at least in how it has done so traditionally. It illustrates a mutual dependence between the social field and the formal organization. The way the teaching practice is organized and the formal role of the students work *because* they have access to the social field and the informal functions. When social activity is reduced to a minimum, interaction, and motivation are affected, and formal practice also becomes less functional.

Where the formal practice does not extend to, or does not work ideally, the informal social field can usually compensate. For example, by insecure students asking questions to fellow students rather than asking questions in the lecture or by informal colloquium groups making up for the need for the open discussion one needs for professional development in those subjects where one does not have seminars. In addition, the social field is thought to be crucial for the holistic education and socialization processes that students go through. It is in social interaction that one acquires the culture that accompanies the knowledge and competence that the students can apply in their own identity construction, as well as social and professional development.

During the coronavirus pandemic, several changes were introduced to the technical structures and practices that resulted in a partial obscuration of the social field. Under these altered circumstances, the lack of social interaction and access to the university as a meeting place was evident. Therefore, it is not surprising how social interaction dominates sensemaking in the informants’ statements.

Our study would also have some theoretical implications. The study shows the importance of combining sensemaking theories with self-determination theory. The role of social interaction in teaching is made visible though our analysis of sensemaking and sensebuilding during the period of lockdown. The term social induced motivation is brought forward to our attention by understanding how the students and staff interpret the situation and change their behavior. This was by example shown when the students tried to establish colloquium groups even when they lack the social arenas to establish such groups. The sensemaking process activated the students to take action due to ‘lack of social communities’, being one of the psychological needs defined by the self-determination theory.

## Limitations

Although the aim of this study is not to provide evidence for the importance of socially induced motivation in learning, the study is enhancing the understanding of its role and importance. Limitation in this study could be related to the study context of only studying two bachelor programs in one western country. On the other side, the study could also have given even more attention to the particular experience from each of the student involved and by this have enriched the study. In our study we aimed to balance this by combining both the students sensemaking as well as organizational handling.

Our investigations were performed during the pandemic, this could affect the perspectives of the informants given a more negative descriptions of the situation compared to studies performed after the pandemic. Other studies have shown a higher degree of reported lockdown fatigue when students are in the crises compared to afterwards (Hassan et al., 2022).

## Conclusions

The period of pandemic and social closure has been demanding for all involved. The academic staff and students in this study emphasized the loss of content when the university as a meeting place and social field was not available. Spontaneous activity in the field, such as conversations during breaks, random meetings in the corridors, and the like, are assigned key functions, such as information sharing and knowledge development. One finding is that some of the students are dependent on being able to build good relationships with fellow students with the opportunity for support, motivation, and internal regulation in smaller student groups. Deficiencies or changes in the social field therefore seem to go beyond motivation. Socially induced motivation is introduced as a current driver and motivator for academic and social activity at the university. The form of motivation is thought to arise because of socialization as a basic function and mechanism in the field, as well as the internalization of common knowledge development. The study concludes that the academic side of the university’s projects, including education and research practice, consists of an interdependence between practice in the formal organization and activity in the social field. When the social field and its functions are severely limited during the pandemic, it also has consequences for the effectiveness of the functions of formal practice.

The interdependence between formal versus informal and socially induced motivation in a university education is one of the clearest and most important findings of this study. If digital teaching becomes the standard for all teaching practice, there is a risk that university education will be completely different from what it has traditionally consisted of, especially given that the acquisition of culture and identity in academia is thought to be partly dependent on activity in the social field and the meeting place of the university as such.

As Gagné & Deci (2005) emphasize, social communities enabling students and staff to feel competent and autonomous in the work are particularly crucial for motivation. The social context is in our study presented as fundamentally valuable and crucial for the socialization process. The implication of our findings for the University would therefore be to carefully sort out the activities to efficiently provide digital teaching while at the same time shield and formalize social arenas for teaching and learning.
